# On the Use of Movement-Based Interaction with Smart Textiles for Emotion Regulation

**DOI:** 10.3390/s21030990

**Published:** 2021-02-02

**Authors:** Mengqi Jiang, Vijayakumar Nanjappan, Martijn ten Bhömer, Hai-Ning Liang

**Affiliations:** 1 Department of Computing, Xi’an Jiaotong-Liverpool University, Suzhou 215123, China; Mengqi.Jiang@xjtlu.edu.cn; 2 Center for Ubiquitous Computing, University of Oulu, 90570 Oulu, Finland; Vijayakumar.Nanjappan@oulu.fi; 3 Faculty of Science and Engineering, University of Nottingham Ningbo, Ningbo 315000, China; martijn.ten.bhomer@nottingham.edu.cn

**Keywords:** movement-based interaction, emotion regulation, feedback mechanism, smart textiles, interactive textiles

## Abstract

Research from psychology has suggested that body movement may directly activate emotional experiences. Movement-based emotion regulation is the most readily available but often underutilized strategy for emotion regulation. This research aims to investigate the emotional effects of movement-based interaction and its sensory feedback mechanisms. To this end, we developed a smart clothing prototype, E-motionWear, which reacts to four movements (elbow flexion/extension, shoulder flexion/extension, open and closed arms, neck flexion/extension), fabric-based detection sensors, and three-movement feedback mechanisms (audio, visual and vibrotactile). An experiment was conducted using a combined qualitative and quantitative approach to collect participants’ objective and subjective emotional feelings. Results indicate that there was no interaction effect between movement and feedback mechanism on the final emotional results. Participants preferred vibrotactile and audio feedback rather than visual feedback when performing these four kinds of upper body movements. Shoulder flexion/extension and open-closed arm movements were more effective for improving positive emotion than elbow flexion/extension movements. Participants thought that the E-motionWear prototype were comfortable to wear and brought them new emotional experiences. From these results, a set of guidelines were derived that can help frame the design and use of smart clothing to support users’ emotional regulation.

## 1. Introduction

Emotion plays an essential role in human behavior and is often portrayed as an automatic impulse elicited by internal or external stimuli. The instantiation of an affective state necessarily involves alterations in the body’s physiology [1,2]. These changes in people’s physiological systems hold the potential to impact physical health directly. Physiological responses meant to be adaptive in the short-term can lead to maladaptive outcomes in the long-term if not regulated correctly [3]. Digital technologies for mental health have great potential but are still in their early stage [4]. Further research is important given the growing incidence of cases, particularly in our current challenging times.

Effective emotion regulation is crucial to both physiological and psychological well-being and social functioning [5]. Emotion regulation consists of people’s active attempts to manage their emotional states and its general coping strategies proceed from three aspects: (1) attention, (2) knowledge/cognition, and (3) body [6]. In the last few decades, there has been growing interest in researching person-oriented emotion regulation. People’s bodies are regarded as an emotion-biasing system, and non-invasive methods for regulating emotions include meditation, mindfulness training, controlled breathing, and progressive muscle relaxation [6]. Moreover, as the development of computing technologies has made it possible for them to be closer to the human body, these emotion regulation strategies have been augmented with multi-sensory wearable devices, including smart textiles. What has been largely ignored is that body motions, including bodily postures, voluntary and involuntary motor movements, can also affect emotions. The potential of using body movement-based interaction for emotion regulation remains relatively unexplored [7,8,9].

Smart textiles have the advantage of being close to the body, can adapt to external stimuli, and monitor physiological signals and movements at any time and in an uninterruptible manner [10]. Recently, there has been increasing interest in the design of emotion-related smart textiles [11,12,13,14]. Some researchers use smart textiles to monitor emotion-related physiological data and then transfer wearers’ emotional states into various sensory feedback mechanisms for further emotion expression, communication, or self-reflection [15,16]. Others have tried to influence emotions through feedback modalities placed in the smart textiles [17]. We believe that smart textiles have the potential to regulate people’s emotions using their body movements when encouraged to do so at the right time and giving the proper sensorial feedback. However, what remains unclear is how we can utilize and leverage body movements to design interactive textiles to help people regulate or improve their emotional states.

This research aims to explore whether movement-based interaction supported by smart textiles could be used for emotion regulation and to identify important factors that influence their design. To this end, we developed a smart textile prototype and conducted an experiment with it to investigate the following:

1. *Body movements and emotions*. The experiment aims to find out which body movement has a positive impact on users’ emotional state(s);
2. *Feedback mechanisms*. Because the type of augmented sensory feedback may also have an impact on the users’ emotional state(s), the experiment aims to explore their preferences on feedback mechanisms when eliciting specific movements;
3. *Movement and feedback mechanisms*. We also want to determine if there is an interaction effect between movement(s) and feedback mechanism(s);
4. *Emotion assessment*. Finally, we want to determine whether participants’ self-emotional assessment is consistent with their emotional expressions when evaluating the prototype.

To do this, we developed a wearable full sleeve t-shirt, which we named as E-motionWear. It can detect four body movements (elbow flexion/extension, shoulder flexion/extension, open and closed arms, neck flexion/extension) and provide three basic feedback mechanisms (audio, visual, and vibrotactile) to motivate users to perform these movements. Previous psychological literature shows that these four body movements can regulate emotion in an effective manner [7,8,18,19,20,21,22,23,24]. In this paper, we report the results of our experiment and offer a new perspective on emotion regulation through movement-based interactive textiles.

## 2. Related Work

In this section, we review the literature related to and that has influenced our work. We begin by discussing movement-based strategies for emotion regulation. Next, we investigate the connection between emotions and feedback mechanisms. Finally, we discuss prior research on smart textiles in emotion regulation to better identify the importance of our work.

### 2.1. Emotion Regulation and Body Movements

Much of the literature on movement-based strategies for emotion regulation comes from psychology. Darwin and James-Lang [25,26] cultivated the initial research on the relationship between movement and emotion. Damasio [27] further claimed that emotions are produced by transferring the body’s current state to the brain through interoceptive and proprioceptive senses. This research led to the somatic marker hypothesis, which posits that the emotion and corresponding body changes are associated with specific situations and their past outcomes. One crucial implication proposed by Damasio is that one’s feelings could be regulated through deliberate changes in one’s posture, movements, and consequent physiological responses [18,27].

The strategy based on Damasio’s work has been demonstrated experimentally. Prior studies reported that some exercises, such as Tai-chi, Yoga, and Qigong, are very useful in emotion regulation [28]. For instance, Punkanen et al. [29] investigated the use of body and movement-based therapy intervention to treat depression among working-age adults. Brinol et al. [30] showed that an enacted affective body position biased people’s attitudes towards the enacted emotion. Early work by Cacioppo et al. [24] observed that arm gestures performed during the evaluation of neutral objects affectively biased their appreciation. Arm gestures that are generally associated with an approach-motivational orientation led to a more positive judgement of the neutral objects than arm gestures associated with an approach-withdrawal orientation. Using an integrated approach, Irene and Gary demonstrated that even a relatively generic pose of a closed or open body posture could evoke emotional responses [19]. Similarly, the research of Casasanto and Dijkstra [23] showed that moving objects with upwards facing hands facilitated the retrieval of positive emotions, whereas downwards hands led to faster retrieval of negative emotions. Rooij and Jones’s research on creativity indicates that certain arm gestures can affect people’s emotions and enhance creativity [20,21,22]. Shafir [18] concluded that the vertical upward movements, expanded torso or limb motions characterized happy actions. In contrast, sad movements were characterized by a collapsed or slumped torso motion. In other studies, she has applied Laban Movement Analysis to identify the responding movement for specific emotion enhancement [9]. In short, these studies highlight the efficacy of body movements, even simple ones, on regulating different aspects of people’s emotions.

Game-based research also has explored motions to generate emotions in players [31]. For example, Zangouei et al. [32] designed the interactive system EmoRoll, which requires a pair of users to generate specific emotions through body movements or expressions to solve the riddles in games. The riddles can be solved using different emotional states such as dancing happily, being scared and relaxed breathing. Their results showed that physical movements involving user’s body movements helped build their emotional process for intense narrative games. Studies by Melzer et al. [33] and Isbister [34] found that games that encourage body movement lead to higher emotional arousal levels than those that use a standard controller.

In this work, because the connection between clothing is an inseparable part of what people wear, we want to study how to design movement-based interactive wearable artefacts that can help people regulate their emotions. Simultaneously, interactive clothing can be used to sense body movements continuously and, as technology advances, inconspicuously.

### 2.2. Feedback Mechanisms and Emotions

Visual, auditory, or haptic feedback can guide user behavior. For example, by increasing the tempo or volume of music, one can encourage more intense body movement or, by causing a controller to vibrate when movement accuracy falls below a particular threshold, it is possible to elicit a rapid response [35,36].

In recent years, increasing research is available on sensory feedback mechanisms of embodied and tangible interaction and their effect on our feelings [35]. Previous studies have examined the association between emotion and feedback mechanisms, like how thermal stimuli are related to human emotions and how we can communicate emotions with thermal feedback [37,38,39]. Yoshida et al. [40] suggested that it is possible to artificially manipulate emotional states using visual feedback of deformed facial expressions in real-time. Macdonald et al. [41] observed affective responses to emotionally resonant vibrotactile stimuli that evoke real-world sensations. Vibrotactile properties such as vibration duration and pattern were also used in information and emotion communication between couples [42]. The effect of sound on emotion and behavior was also demonstrated by Bresin et al. [43]. By altering the sound produced by a person’s walking steps, they were able to alter the person’s perception of the walking surface material. In Tajadura-Jimenez et al.’s work [44], it is shown that using special shoes embedded with microphones to capture and deliver back to people the altered sound of their footsteps, they could control people’s perception of their bodies and alter their walking behavior and emotional states. Overall, these studies suggest that feedback mechanism plays a role in people’s emotional states.

### 2.3. Smart Textiles and Emotions

Smart textiles provide close contact with the wearers’ skin and can sense and communicate the wearer’s stimuli, conditions, and body movements. Besides, smart textiles exhibit significant benefits in terms of usability for long-time monitoring and provide maximum comfort with few to no constraints. Valenza et al. [45] investigated the use of Electrodermal Response changes for emotion recognition using textile electrodes on a fabric glove. Similarly, Wu et al. [46] developed a wearable textile-based emotional management system using a heart rate variability (HRV) biofeedback system. They reported that real-time HRV biofeedback is quite efficient in cases of negative emotion.

Likewise, Zhou et al. [47] explored how pressure-sensitive smart textiles can help monitor people’s emotional states through changes in facial expressions. They proposed the use of textile pressure mapping arrays integrated into a headband to capture the forehead muscle movements. Their Expressure system achieved 82% accuracy to recognize three eyebrow movements. On the other hand, Eli et al. [17] designed ThermalWear, a wearable thermal display using neoprene wetsuit diving tops, to explore how thermal displays can induce emotions. They used Peltier elements as primary thermal actuators on the inner side of the upper chest part of the diving vest. The Peltier element is connected to the heat sink, which is placed on the other side of the vest through a hole with thermal glue. They found that the perception of voice messages can be augmented with thermal stimulation. Gravina and Li [48] developed a smart cushion to detect fundamental seated gestures relevant to emotion regulation. Their system consists of a force sensing-resistor deployed on the cushion and two inertial measurement units (IMUs) worn on the user’s wrists. The pressure and motion signals are used to recognize the following everyday emotion-relevant activities: (1) interest, (2) frustration, (3) sadness, and (4) happiness. The smart cushion is used to accurately recognize five different sitting postures (proper sitting, leaning right/left, and leaning forward/backward). The upper limb gestures (arms crossed, arms raised, arms straight down, and hands holding the head) are captured using the wrist-worn IMUs.

Wang et al. [49] investigated the relationship between human emotions and smart textiles to design interactive clothing for couples. Their prototypes consist of a cold-proof liner with cotton filling, thermoplastic polyurethane, LED ribbons, an ultrasonic sensor, and a single-chip microcontroller. They used distance as a trigger for interaction. Thus, the LEDs would illuminate when the distance was reduced between the couples wearing the prototypes. The Kansei evaluation proved that the smart textiles correlated well with human emotional and expressive patterns. Lugt and Feijs [11] investigated the use of smart textiles for stress reduction solutions with haptic feedback. To do that, they have embedded visually appealing soft actuators in everyday garments using embroidery of conductive yarns in triangular shapes. They created artificial goosebumps using both conductive and embroidery threads. Their personalized patterns can be integrated into the users’ favorite clothing design, which can relieve stress by reminding them to adjust their breathing patterns. Another design case is an interactive shawl that reacts to users’ emotional arousal to help them reflect on their own emotions [50]. Users’ emotional variations were embodied with light/vibration feedback on the shawl and led the users to regulate their emotion through actions such as poking light/vibration bubbles on the shawl. Their preliminary results showed the potential of daily wear smart textiles on emotion regulation [50].

In short, while research has indicated what types of movement can stimulate emotions and how different types of feedback mechanisms affect emotions [18,19,28], there are still unknowns regarding what type of movements are more effective in stimulating positive emotion, how feedback mechanisms affect emotion in the process of motivating movement, and whether there is an interaction effect between these two factors. This study specifically explores the use of a wearable smart textile that can capture different body movements and provide sensory feedback mechanisms to the user.

## 3. E-motionWear

In this section, we discuss the selection of body movements and the design of feedback mechanisms supported by the E-motionWear prototype, followed by the build process of this smart t-shirt prototype.

### 3.1. Movements

**Figure 1:**
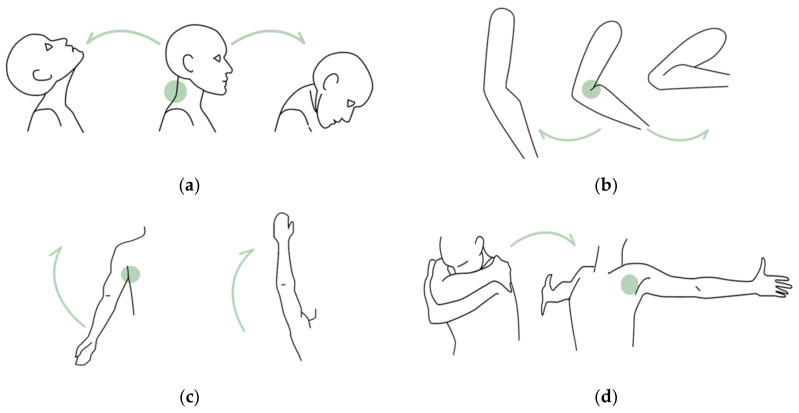
The body movements detected using our prototype: (**a**) neck flexion/extension; (**b**) elbow flexion/extension; (**c**) shoulder flexion/extension; and (**d**) open and closed arms. Green dots indicate the sensors’ position.

This study explored the scenario where a textile-based emotion wear t-shirt would be available to users. Initially, we reviewed the literature to identify the body movements that impact human emotions [7,8,18,19,20,21,22,23,24]. We identified the flexion and extension movements that affect the angle between two parts of the body. Subsequently, we identified the following body parts where these movements can occur: (a) neck, (b) elbow, (c) shoulder, and (d) arms (see Figure 1). While open and closed arms and neck flexion movements belong to the torso expanding motions, the elbow and shoulder flexion/extension belong to the upward-opening body movements. These movements are thought to contribute to enhancing positive emotions [7,8,18,19,20,21,22,23,24]. Moreover, the smart t-shirt covers the upper body allowing the users to conveniently perform these movements in any posture (such as standing or sitting).

### 3.2. E-MotionWear Sensing

**Figure 2:**
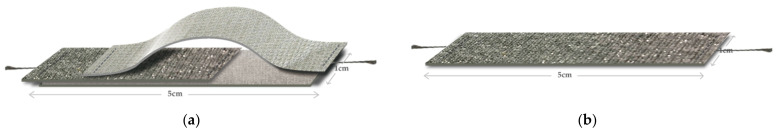
Two types of fabric sensors used in our prototype: (**a**) Neck flexion/extension fabric sensor, made of two kinds of conductive fabrics; (**b**) Arm, elbow, and shoulder movements’ fabric sensor, made of conductive knitted elastic fabric.

**Figure A1:**
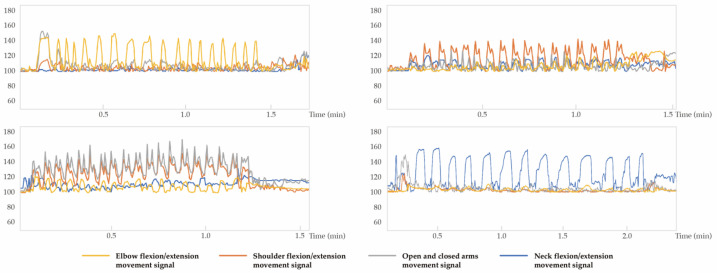
The processed signals of the four movements from the textile sensors.

Two types of fabric sensors were developed to capture these body movements precisely, one with a conductive (made of 18% silver) knitted elastic fabric and the other with two pieces of conductive fabrics. We used three conductive knitted fabric sensors to capture the elbow, arm, and shoulder movements. The change in resistance with the increase of tensile strength reflected the bending range of the corresponding movement. Similarly, we used a fabric sensor (made of two pieces of conductive fabric) in the back of the neck to capture neck flexion movements. The conductive fabric with high resistance (400 ohm) was placed above the low resistance fabric (50 ohm). A conductive thread was used to secure the connection. One end of the conductive thread was connected with the high resistance fabric, while the other end was placed between the two conductive fabrics. The contact range between the low and high resistance fabrics would increase when the head is raised. The current would pass through the low resistance fabric more, thus changing the current on two sides of the sensor and serve as an indicator of the head raising movement (see Figure 2). Figure A1 in Appendix A shows a screenshot of the signals from the four movements captured via the textile sensors.

### 3.3. Feedback Mechanisms

Three different sensory feedback mechanisms were implemented in our prototype: audio, visual, and vibrotactile. Four LED lights with different colors and brightness were used to provide visual feedback to the users. For the vibrotactile feedback, we used four motors, which corresponded to four body movements. The movement amplitude was mapped to the vibration intensity of the motor. Similarly, the Bluetooth transmission of the BLE Nano was used to send movement signals to a mobile app to play audio feedback. Four kinds of audios, each corresponding to a particular movement, were mapped to the phone App volume.

### 3.4. E-MotionWear Implementation

**Figure 3:**
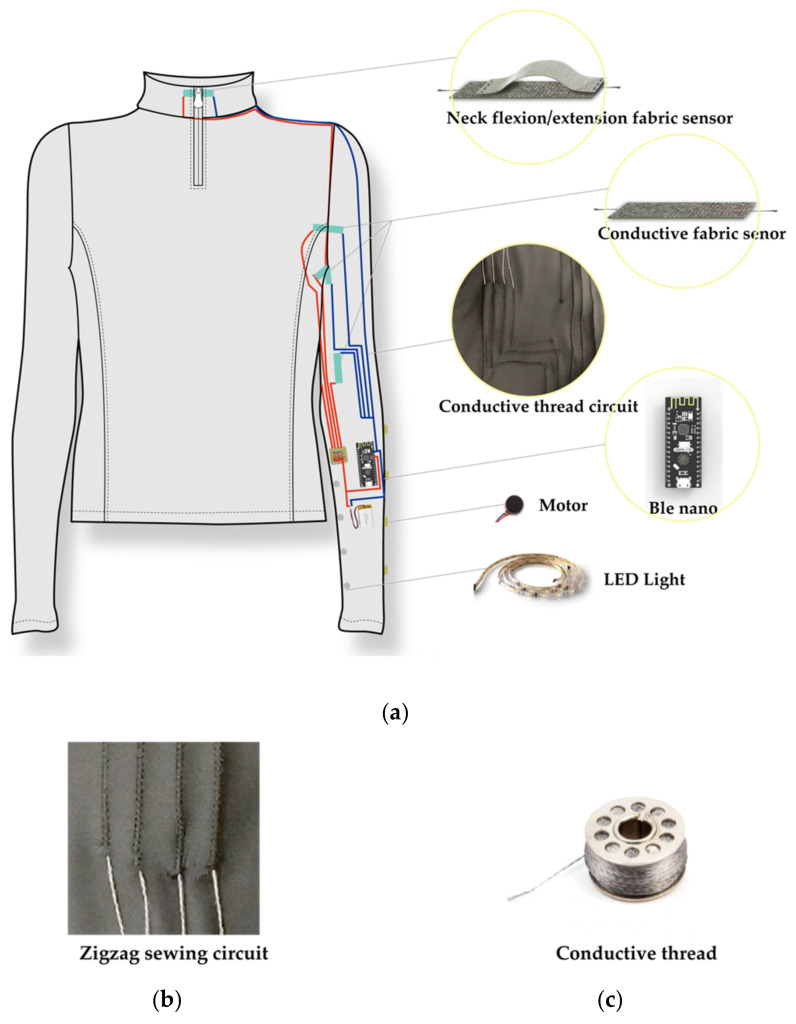
(**a**) Illustrative image of our E-motionWear t-shirt with details of the components; (**b**) Zigzag sewing to protect the conductive thread circuit; and (**c**) Stainless conductive thread employed in our prototype.

**Figure 4:**
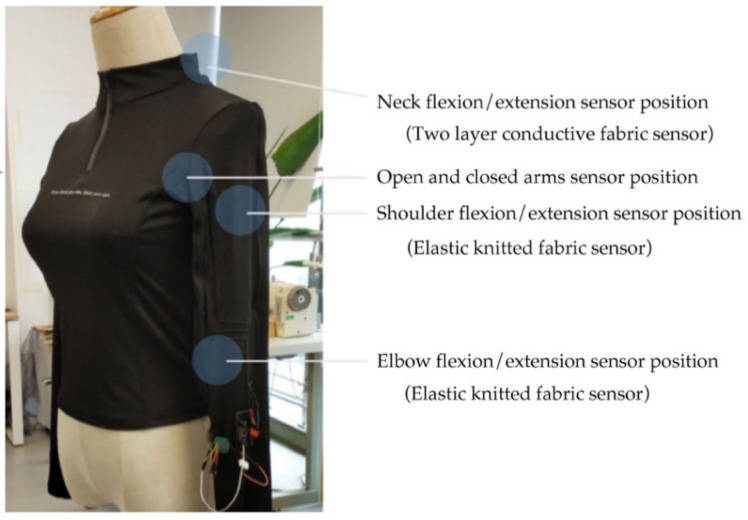
The female version of the E-MotionWear prototype and placement of the different sensors.

We produced two versions of the prototype, one for male and the other for female users. All prototypes were made of highly stretchable jersey fabric, of medium size, and had the same color, electronic devices, and components. A flexible stainless-steel thread was embedded in the prototype as the conductive wire to secure the connection with high conductivity. We used non-conductive thread in a zigzag sewing pattern to fix the conductive thread on the stretchable knitted textiles. The conductive thread inside the zigzag stitch played a specific insulation effect and would move flexibly with the elastic fabric’s stretch without affecting the clothing’s shape (see Figure 3 and Figure 4).

## 4. User Evaluation

The aim of the user evaluation was to explore: (1) which body movements had a significant effect on users’ emotional state; (2) which type of feedback mechanisms were preferred to elicit particular movements; and (3) whether there was a relationship between users’ emotional state and facial expressions. This study used a 4 (Body Movements) × 3 (Feedback Mechanisms) two-way repeated factorial design. Each participant received all experimental combinations. The order of presentation was counterbalanced using a Latin Square design.

### 4.1. Participants

Fourteen student volunteers (5 males, 9 females) from a local university were recruited from social media platforms for the experiment. They all came from its department of industrial design or architectural design; 4 of them were graduate students, and 10 were undergraduate students. They aged between 18–25 years (M = 21.5, SD = 2.10). None of them has prior experience with smart textiles.

### 4.2. Measures

This study employed the following measurements to evaluate the performance of the prototype and understand users’ preferences and facial reactions to the body movements:

- Participants’ subjective emotional feelings were collected using the Self-Assessment Manikin (SAM) [51] questionnaire;
- The AffdexMe App [52] was used to capture facial emotions. This study considered the following AffdexMe facial emotions: Disgust, Joy, Sadness, Surprise, Engagement, and Valence;
- Our prototype’s wearable comfort was evaluated using the Comfort Rating Scale (CRS) [53], a value between 0 to 20.

### 4.3. Apparatus

**Figure 5:**
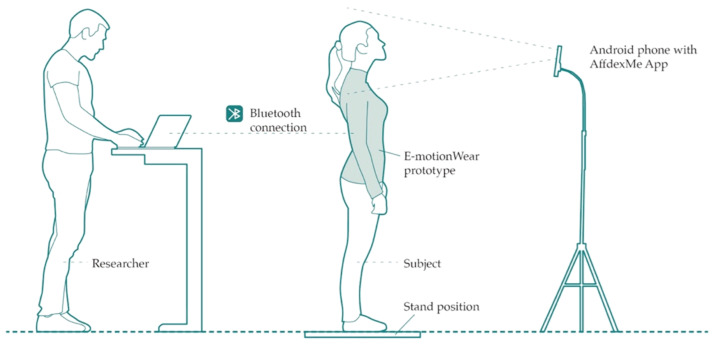
Illustration of the experiment apparatus and set up.

The study was conducted in a laboratory, where the participants wore the E-motionWear as depicted in Figure 5. The instructions were given verbally to each participant. An Android phone was used to capture the facial expressions of the participants.

### 4.4. Procedure

**Figure 6:**
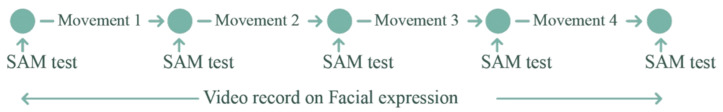
SAM tests that were used in the experiment process.

At the beginning, participants were introduced to the purpose of the study. After explaining the process of the experiment, each participant signed the experimental consent form to participate in the study. Then, they were given a suitable E-motionWear prototype to wear in a separate empty room. All participants were given sufficient time to learn and familiarize themselves with the movements. One of the researchers asked them to distinguish the audio, visual, and vibrotactile units that corresponded to their body movement. This step ensured the functionality of the prototype for each participant. Next, participants were asked to perform each movement 15 times. A three minute break was given in between every movement, but more could be given when requested. Each participant was asked to complete the SAM questionnaire before and after performing each movement (see Figure 6). All participants were requested to stand in a pre-determined location to record their facial emotions. The camera on the smartphone mounted on a tripod was used to capture the users’ facial expressions. We encouraged them to freely express their feelings and opinions during the experiment, and their verbal feedback was audio recorded during the experiment for further analysis. In the end, they were asked to complete the Comfort Rating Scale. The whole process took around 45 min for each participant.

## 5. Results

The main measurements for evaluating the performance of E-motionWear were the confusion matrix under three types of feedback mechanisms. We also performed an analysis of users’ self-assessment emotions and facial emotions in SPSS using a two-way repeated-measures ANOVA with post-hoc tests if necessary. The statistical significance was set at *p*  <  0.05. F and M represent the gender of the participants in our analysis. The numbers after the letters F and M (e.g., F1 or M1) represent the order of each participant.

### 5.1. E-MotionWear Performance Assessment with Confusion Matrix

**Figure 7:**
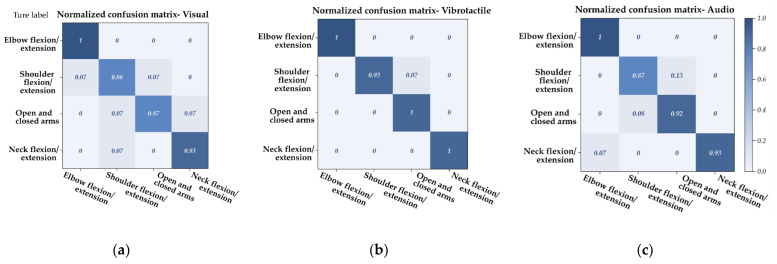
The normalized confusion matrix results for movement recognition of the E-motionWear prototype: (**a**) visual, (**b**) vibrotactile, and (**c**) audio feedback mechanisms.

Figure 7 shows the confusion matrices indicating the number of times that E-motionWear recognized each body movement for the three feedback mechanisms. We used the F1-Measure (the weighted average value of Precision and Recall) results to show the performance of our prototype. The F1-Measure of vibrotactile and audio feedback mechanisms was 0.983 (Figure 7b) and 0.93 (Figure 7c), respectively, while the visual feedback attained 0.912 (Figure 7a). Several participants mentioned that visual feedback is distracting to follow during body movements. In more detail, the neck flexion movement achieved an F1-measure of 0.965, 0.976 for the elbow flexion movement, and 0.917 for the shoulder flexion movement. While the open and closed arm movement’s F1-measure was 0.907. While still having high accuracy, the participants’ shoulder movements can exhibit more significant variations, so the sensors placed around the shoulder showed less accuracy and recall than the elbow and neck movements.

### 5.2. Self-Assessment Manikin (SAM) Analysis

We used SAM ratings to examine the effect of different body movements and feedback mechanisms in users’ emotional states.

#### 5.2.1. SAM-Valence

We performed ANOVA tests on emotional valence with feedback mechanisms (A3 audio, A1 visual, A2 vibrotactile) as variable A and movements (B0, B1, B2, B3, B4) as variable B. B0 represents the initial movement (standing upright in the required position), and B1 elbow flexion, B2 shoulder flexion, B3 open arms and B4 neck flexion movements. There are 15 combinations (A × B = 15) in total. Each participant repeated all 15 combinations (N = 14).

**Figure 8:**
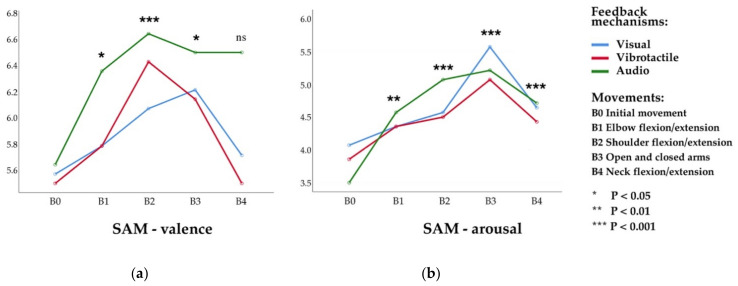
Estimated Marginal Means of SAM questionnaire data when participants perform under different movements and feedback mechanisms. (**a**) SAM valence—the intrinsic positive or negative valence of their emotional feelings; and (**b**) SAM arousal—the emotional state of being awakened.

The results show that the main effect of the movement was significant (*F*_(2.597,33.763)_ = 3.251, *p* = 0.04, η2 = 0.2), and the main effect of the feedback mechanism was marginally significant (*F*_(2,26)_ = 2.806, *p* = 0.079, η2 = 0.178). No effect of condition or interaction between the two was observed. The results suggest that participants’ movement was a reliable predictor of emotional valence, more preferentially than feedback mechanism (see Figure 8a).

Post-hoc pairwise comparisons on movements revealed that participants’ emotional valence was significantly higher for elbow flexion, shoulder flexion, open arm movements than the initial condition (B1&B0, *p* = 0.018; B2&B0, *p* < 0.001; B3&B0, *p* = 0.023). Shoulder flexion movement was significantly higher than elbow flexion movement (*p* = 0.02, mean difference 0.405, (95% CI: 0.501–1.118)). Although the feedback mechanism’s main effect was marginally significant, post-hoc pairwise comparisons on the feedback mechanism showed that the audio mechanism was also marginally significant compared to the visual mechanism (*p* = 0.063, mean difference 0.457, (95% CI: 0.029–0.944)).

#### 5.2.2. SAM-Arousal

For emotional arousal, we found that the main effect of the movement was significant (*F*_(2.488,32.350)_ = 8.924, *p* < 0.001, η2 = 0.407). Post-hoc pairwise comparisons on movements revealed that participants’ emotional arousal was significantly higher for all four movements than the initial condition movement (B1&B0, *p* = 0.002; B2&B0, *p* < 0.001; B3&B0, *p* < 0.001; B4&B0, *p* < 0.001). In particular, open arm movement was significantly higher than elbow flexion and neck flexion movements (B3&B1, *p* = 0.021; B3&B4, *p* = 0.039) (see Figure 8b).

#### 5.2.3. SAM-Dominance

The emotional dominance data shows no statistical significance. Overall, in the aspect of emotional valence and arousal, body movements showed significant differences, while the feedback mechanism revealed marginal significance on emotional valence.

### 5.3. Facial Expression Analysis

**Figure 9:**
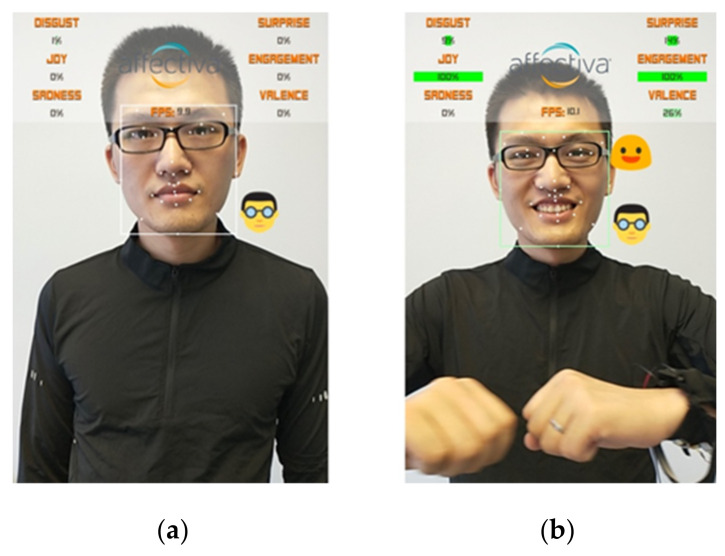
An example of the output of the AffdexMe App on a facial expression during the experiment: (**a**) initial position and (**b**) while performing the open arm movement.

This study used AffdexMe [52], a mobile application for facial emotion analysis. The facial expression validity of Affdex software was proven to be comparable to facial electromyography measures [54], and no electrodes were needed. We collected data for the following six kinds of emotion: Joy, Surprise, Sadness, Disgust, Valence, and Engagement. Based on the video recordings, we counted the number of times each emotion appeared in the participants’ facial expression when they performed specific movements. Except for the neck flexion, when the head moves up, the participants’ facial data cannot be recorded; the other movements’ facial emotion data can be computed—the frequency of Joy, Surprise, positive Valence, and Engagement data used for further analysis. Figure 9 shows the use of the AffdexMe app to capture participants’ facial expressions during the experiment.

#### 5.3.1. Joy

**Figure 10:**
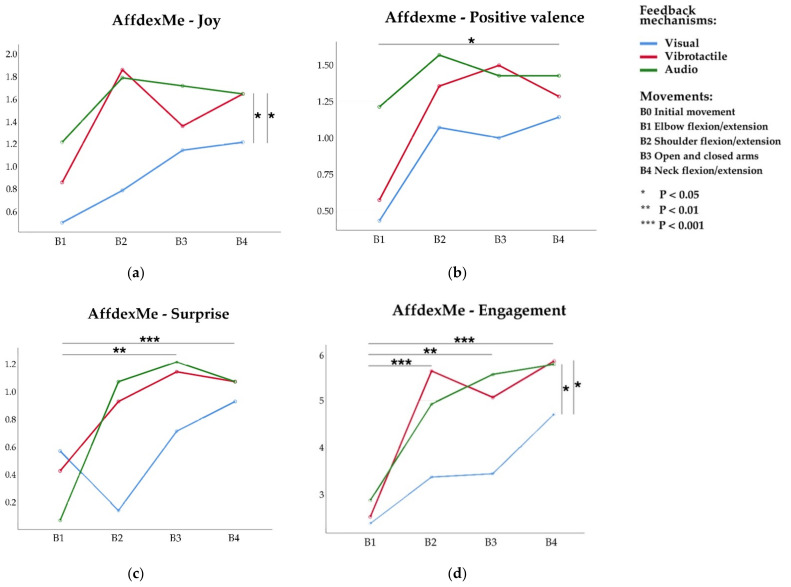
Estimated Marginal Means of the AffdexMe facial data when participants perform under different movements and feedback mechanisms. (**a**) Joy—the frequency of happy emotion during the movement performance; (**b**) Positive valence—the frequency of positive emotion; (**c**) Surprise—the frequency of surprise emotions; and (**d**) Engagement—the frequency of emotional engagement.

An ANOVA on Joy data revealed a significant main effect for feedback mechanism *F*_(2,26)_ = 3.888, *p* = 0.033, η2 = 0.230 (see Figure 10a). There was no significant interaction between movement and feedback mechanism. Post-hoc pairwise comparisons on the feedback mechanism revealed that participants had experienced Joy more frequently in both the audio and vibrotactile mechanisms than the visual (A3&A1, *p* = 0.034; A2&A1, *p* = 0.042).

#### 5.3.2. Surprise

The main effect of the movement was found to be significant for Surprise *F*_(3,39)_ = 5.706, *p* = 0.002, η2 = 0.305 (see Figure 10c). Post-hoc pairwise comparisons on the feedback mechanism revealed that participants had experienced Surprise more frequently in open arm and neck flexion movements than the elbow flexion (B3&B1, *p* = 0.006; B4&B1, *p* < 0.001).

#### 5.3.3. Positive Valence

There was no statistically significant difference for the main effect of the feedback mechanism. However, we found marginal statistical significance for the main effect of the movement *F*_(3,39)_ = 2.567, *p* = 0.068, η2 = 0.165 (see Figure 10b). The pairwise comparisons show that the open arm movement exhibited more positive Engagement than the elbow flexion movement, *p* = 0.034, mean difference 0.548, (95% CI: 0.049–1.046).

#### 5.3.4. Engagement

The two-way repeated-measures ANOVA on Engagement revealed a significant main effect for movement *F*_(3,39)_ = 7.689, *p* < 0.001, η2 = 0.372 and a significant main effect for feedback mechanism *F*_(2,26)_ = 4.303, *p* = 0.024, η2 = 0.249. There was no significant interaction between the two (see Figure 10d). Post hoc pairwise comparisons on feedback mechanism with LSD also revealed that participants performed Engagement more frequently for the audio and vibrotactile mechanisms than the visual (A3&A1, *p* = 0.011; A2&A1, *p* = 0.044). For the movement variable, elbow flexion, open arms, neck flexions exhibited more Engagement than the shoulder flexion (B2&B1, *p* = 0.001; B3&B1, *p* = 0.004; B4&B1, *p* < 0.001).

### 5.4. Wearable Comfort of E-MotionWear

**Figure 11:**
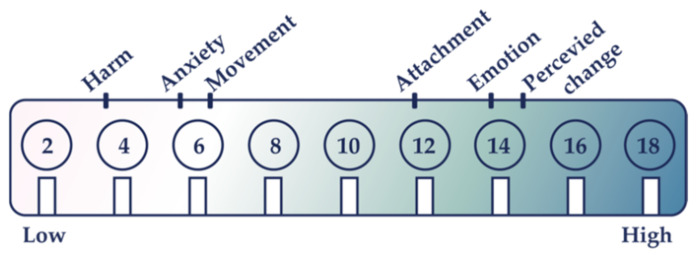
The mean values of the six elements of comfort rating scales for E-motionWear.

To assess the wearable comfort of the E-motionWear prototype, we asked each participant to rate the prototype in the following six attributes of wearable comfort: Emotion, Attachment, Harm, Perceived Change, Movement, and Anxiety using the Comfort Rating Scales (CRS) [49]. Figure 11 shows the mean CRS scores for E-motionWear. The score ranges from 0 to 20. The highest CRS scores were received for the Perceived Change (14.6 ± 4.0) followed by the Emotion (13.9 ± 2.1) and the Attachment (11.7 ± 4.2) elements. The Harm (3.5 ± 4.0) element received the overall lowest scores for our prototype. Our participants mentioned that they felt comfortable wearing our initial version of the prototype during the evaluation study. They also further mentioned that the E-motionWear did not affect or inhibit their body movements. The comfort level relates to individuals’ body shape, and some of them were not so used to wearing tight sportswear but did not complain too much.

### 5.5. Participants’ Feedback

All the participants felt comfortable wearing the E-motionWear prototype and expressed their interest in wearing a smart t-shirt in the near future. Before the experiment, few participants were worried that the conductive thread would pass some electricity to them. However, after some clarification, they were assured that nothing like that would happen. During the interview, participants mentioned that they preferred vibrotactile and audio feedback mechanisms than visual. As F1 mentioned, it was not convenient to see the visual effect while performing the body movements. In particular, M5 mentioned that the visual effect was distracting. Three participants (2 Males) expressed their preference for the vibrotactile feedback as they could feel this tactile sense directly on their body. Notably, several participants felt pleased with the audio feedback. They further mentioned that they would like to see their body movements turn into their favorite music. Some participants even tried to produce melodies with their bodies.

Participants’ feelings on each movement were varied. Overall, they expressed a higher preference for shoulder flexion and open arms movements. In particular, F5 mentioned that she felt like these movements opened her body and mind. M1, F8, M3 said that though shoulder flexion was convenient to perform with E-motionWear, they felt tired after continuously performing it several times. The preference for neck flexion appeared to depend on the user’s cervical spine health condition. For instance, F1 stated that she used to keep her head down for a very long time during the study. Now she felt much more comfortable and positive when raising her head upwards after using the prototype. Likewise, M4 said that neck extension movement made him feel stronger and more confident. On the other hand, F2 felt uncomfortable with neck flexion movement due to her vertebra problems.

Several participants mentioned that they did not have much particular feeling about elbow flexion movement; they thought elbow movement’s amplitude was not large enough to affect their feelings. Moreover, it seemed that whether participants exercised regularly or not affected their preferences. For example, M1 mentioned that he had the experience of teaching Tai-chi, and M5 had three years of Yoga teaching experience. Both of them performed the required movements more precisely and were more sensitive to movement-based interaction and proprioception.

## 6. Discussion

### 6.1. Experimental Evidence

The present study was designed to determine and compare the factors affecting people’s emotions while performing interactive body movements. Initially, we analyzed the experimental results and validated the functionality of our smart textile prototype. Second, we observed how body movements would impact emotional valence and arousal, especially the open-closed arms and shoulder flexion/extension movements from the SAM results. Third, participants’ facial expression data from AffdexMe APP were analyzed, and both movements and feedback mechanism factors were found to impact users’ facial emotion expression. Then, with the experimental findings of SAM, facial expression, and interview feedback, the participants’ subjective and objective emotional responses were horizontally compared. Although there are differences, specific trends stood out. Finally, the CRS results and feedback were used to evaluate the prototype from its wearability and user perception perspectives.

The results of SAM and AffdexMe analysis indicate that no interaction effect between the movement and feedback mechanism. AffdexMe’s Facial Joy and Engagement data indicate that vibrotactile and audio feedback mechanisms have a significantly better effect than visual feedback; the participants’ feedback also supports this point. From the user feedback, we can infer that this result may be associated with the body shaking during the movement, which is unfavorable for visual perception. The ANOVA results of self-assessed emotional valence indicated that the feedback mechanism’s main effect was marginally significant. The audio mechanism was also marginally significant and more favorable compared to the visual mechanism. In contrast, all three feedback mechanisms did not show a clear difference in the self-assessed emotional arousal level. Thus, the overall evaluation of vibrotactile and audio feedback mechanisms are dominant in many respects; the effect of visual feedback might be limited during movements.

From the SAM ANOVA results of movements, we can see the movement factor had a main effect on both SAM valence and arousal. Almost all movements enhanced participants’ self-assessed emotional valence and arousal, except for the neck movement in emotional valence. In particular, the shoulder flexion movement was significantly higher than the elbow flexion movement in SAM-valence, and open arm movement was significantly higher than elbow flexion and neck flexion movements in SAM-arousal. AffdexMe’s Facial surprise and positive Valence data show that open-closed arms and neck flexion/extension movements are significantly higher than elbow flexion/extension and shoulder flexion/extension movements. Besides, the emotion engagement data show that shoulder flexion/extension, open-closed arms, neck flexion/extension movements are more effective than elbow flexion/extension movements.

Further, facial emotion engagement data show that shoulder flexion/extension, open-closed arms, neck flexion/extension movements are more effective than elbow flexion/extension movements. The interview data suggest that participants prefer shoulder flexion/extension and open-closed arms movements. These findings are also consistent with SAM and AffdexMe facial emotion results. The above results are broadly in keeping with previous studies of body movements, particularly in psychology, which indicate that these four body movements impact people’s emotions [18]. However, no apparent effect was found in the elbow flexion/extension movements. This outcome might be because the elbow movement’s impact on people’s emotions is limited and subtle. The controversial movement is the neck flexion/extension. From participants’ statements, the effect of this movement might be associated with the individual cervical spine health. Taken together, from the results of SAM, facial data, and participants’ feedback, we can draw the conclusion that shoulder flexion/extension and open-closed arms movements showed a more significant impact on emotion.

The CRS data indicated that participants thought the E-motionWear prototype was comfortable to wear, but it also led to a novel experience. This finding was also observed with other wearable products people never experienced before [53]. The majority of participants felt fun wearing the E-motionWear prototype and demonstrated interest in it. As M3 said, there was a significant difference from his regular clothes, and he felt engaged with the prototype because his movements were associated with sensory feedback. F4 said this prototype could motivate her to do more exercise, explaining the high scores of Perceived Change, Emotion, and Attachment of CRS. F6 also mentioned it could be used for the rehabilitation of neck problems.

### 6.2. Design Recommendations

The experiment results have helped us identify the feedback mechanisms that can motivate specific body movements and suitable body movements that can improve positive emotional valence and arousal. These results have implications for embodied interaction and contribute to movement-based interactions for emotion regulation. Below are five design guidelines that have been extrapolated from the results:

- *Easily perceivable and pleasant feedback to motivate movements*. The feedback mechanism should provide a pleasant user experience and informative presentation without confusing the users. Given that the wearer will be in different environments on any given day and doing different activities, it is better to provide users with the option to choose their preferred feedback mechanisms. As stated in [36], there is a need for the personalization of movement-based interaction. However, when using movement-based interactive textiles, visual feedback is not easy to capture, and more consideration could be given to tactile and auditory feedback. Besides, as mentioned in [55], vibrotactile can be noticed only on selected body areas to provide an intuitive correspondence to the movement of the user. The amplitude, frequency, or melody of the feedback mechanisms will also affect the user, which should be adjusted according to the actual use scenarios.
- *Upper-body movements to promote positive emotions*. It is important to note that not all movements lead to positive emotions. Among the four movements in the experiment, the body-expanding and upwards movements were proven to be more effective in promoting positive emotions. Using the upper body to execute these movements is more comfortable, both sitting and standing. Effective movements mainly focus on arm activities. However, the movements should not be too complicated and be easy to remember for users. As individuals have their own movement preferences, users should be provided with multiple choices instead of a single movement interaction in a wearable system. Movements should be designed according to their use scenario and users’ physical conditions. Excessive and improper exercise may cause physical fatigue and injury. More attention should be paid to protection, such as the neck movements.
- *Favour fabric sensors*. As [56] concludes, the measurement of video, optical, and accelerometer-based body motion analysis systems are limited in their applicability. In this case, the fabric-based sensor could provide a low-cost solution, especially for long-time movement monitoring. Through fiber materials and structures, multiple fabric movement sensors could be developed to fulfil different requirements.
- *Flexible smart t-shirt for movement detections*. We have several considerations when designing the E-motionWear prototype. The smart textile prototype should be flexible and comfortable for the wearer, fit different body movements and shapes, and able to detect any required movement and its amplitude. Elastic jersey fabrics or knitted fabrics are widely used in smart textiles for monitoring body movements, which have also been proved to be effective, but some users may be uncomfortable wearing them if they are too tight.
- *Aesthetics*. We should hide electronic components with e-textile technologies, like conductive thread, fabric sensor, to avoid unnecessary concerns and anxieties of users, which can be achieved by combing traditional clothing manufacturing techniques into the design of interactive textiles. The intention to use new technologies tends to decline with age [30]. However, the interactive textile interfaces could reduce the obtrusiveness of wearing electronic devices to increase user acceptance from both appearance and psychological aesthetics perspectives.

### 6.3. Limitations and Future Work

There are several limitations to this study, which can also serve to frame future work. Different types of visual, vibration rhyme, audio feedback mechanisms have a differential effect on emotion to some extent [57,58]. However, this experiment aims to study the differences between the three sensory feedback mechanisms in general. We chose the emotional neutral visual, vibrotactile, and audio types to avoid biasing any feedback mechanism. In the future, we aim to address the affective influence of the feedback mechanisms’ parameters.

Besides, the accuracy and validation of the facial expression recognition software may not be consistent and can be affected by various external factors. For instance, a study revealed that iMotions’s FACET module outperforms its AFFDEX module, while new customers of iMotions can only use AFFDEX [59]. Prior research indicated that iMotions’s algorithms fail to detect non-prototypical emotions, which can be consequential because the compound and/or subtle facial expressions are prevalent [59].

Another limitation is the facial emotion recognition when neck movements are involved. Because of the inherent nature of neck movements, it is challenging to capture reliable facial data, which could result in mispredictions from any software when users flex/extend their necks. Further investigation is needed to determine efficient in-motion facial emotion recognition methods, something beyond the scope of this research, but a direction worthy of further research given the increasing demand for accurate emotion recognition software.

Finally, previous studies regarding the bio-signals for emotion recognition would be worthwhile to validate users’ emotional state, e.g., heart rate variability [60], heart sound signals [61], electroencephalography [62]. The emotional state could also be recognised from movement features [63], as Melzer et al. have been to able to verify, that the Laban Movement Analysis components of movements are associated with emotion recognition. It would be interesting and useful to investigate whether the emotional state could be recognised from the signals of the fabric-based movement sensors (see Figure A1, in Appendix A).

## 7. Conclusions

This research set out to assess the effects of emotion-related factors in the movement-based interactive wearable systems. To achieve this, we built the E-motionWear prototype with low-cost fabric sensors, visual, vibrotactile, and audio feedback electronic components. Multiple analyses from participants’ SAM, facial expressions, and interviews revealed that several specific movements and feedback mechanisms have a better effect on evoking a positive feeling. In general, shoulder flexion/extension and open-closed arms movements made people feel more positive and aroused. The light feedback mechanism is distracting during arm motions, while participants prefer vibrotactile or audio feedback.

This study’s findings suggest that movement-based interactive textiles could be used for emotion regulation if well designed. Participants are generally accepting of wearing interactive textiles. Future studies should consider the actual application scenarios of a movement-based interaction system to regulate emotions. In this experiment, we studied how to affect emotions by motivating movements under laboratory conditions. In real life, people often face more complex emotions and situations. It is worth exploring further if there are any differences in the effectiveness of movement-based interaction on regulating emotions based on different natural settings and investigating its acceptance by users in such environments.

## Data Availability

Data can be made available upon request.
